# The Influence of rs1859168 Polymorphism on Serum Expression of HOTTIP and Its Target miR-615-3p in Egyptian Patients with Breast Cancer

**DOI:** 10.3390/biom11050733

**Published:** 2021-05-14

**Authors:** Omayma O. Abdelaleem, Olfat G. Shaker, Marwa N. AbdelHafez, Noha K. Abdelghaffar, Hanaa M. Eid, Mohamed Zaidan, Abeer A. Khalefa, Naglaa A. Ahmed, Nada F. Hemeda, Othman M. Zaki, Aeshah Ali A. Awaji, Shereen R. Mohammed

**Affiliations:** 1 Department of Medical Biochemistry and Molecular Biology, Faculty of Medicine, Fayoum University, Fayoum 63511, Egypt; ooa00@fayoum.edu.eg (O.O.A.); srk00@fayoum.edu.eg (S.R.M.); 2 Department of Medical Biochemistry and Molecular Biology, Faculty of Medicine, Cairo University, Cairo 11511, Egypt; Olfat.shaker@kasralainy.edu.eg; 3 Department of Medical Oncology, National Cancer Institute, Cairo University, Cairo 11511, Egypt; marwa_nabil_25@yahoo.com; 4 Department of Clinical pathology, Faculty of Medicine, Fayoum University, Fayoum 63511, Egypt; nka00@fayoum.edu.eg; 5 Department of Microbiology and Immunology, Faculty of Medicine, Fayoum University, Fayoum 63511, Egypt; 6 Department of General Surgery, Faculty of Medicine, Fayoum University, Fayoum 63511, Egypt; Mfz00@fayoum.edu.eg; 7 Department of Physiology, Faculty of Medicine, Zagazig University, Zagazig 44523, Egypt; abeerbiomy@zu.edu.eg (A.A.K.); naahmed@nu.edu.sa (N.A.A.); 8 Department of Genetics, Faculty of Agriculture, Fayoum University, Fayoum 63511, Egypt; nfh00@fayoum.edu.eg; 9 Department of Clinical Pathology, Faculty of Medicine, Damietta University, Damietta 34511, Egypt; Drosmanzaki@hotmail.com; 10 Department of Biology, Faculty of Science, University College of Taymaa, Tabuk University, Tabuk 47711, Saudi Arabia; aawaji@ut.edu.sa

**Keywords:** breast cancer, fibroadenoma, rs1859168, HOTTIP, miR-615-3p

## Abstract

Background: Polymorphisms of long noncoding RNAs are lately documented as hazardous factors for the development of numerous tumors. Furthermore, the evaluation of noncoding RNAs has emerged as a novel detector of breast cancer patients. We aimed to genotype the HOXA transcript at the distal tip (HOTTIP) rs1859168 and assess its relationship with the levels of the serum HOTTIP and its target miR-615-3p in patients with breast cancer (BC). Methods: One hundred and fifty-one patients with BC, 139 patients with fibroadenoma (FA), and 143 healthy participants were incorporated into the current study. The genotyping of rs1859168 and the measurements of the HOTTIP and miR-615-3p levels were assessed using quantitative real-time PCR. Results: We revealed a significant association between each of the CC genotypes, C allele, dominant and recessive models, and the increased risk of BC (*p* = 0.013, *p* < 0.001, *p* < 0.001, and *p* < 0.001, respectively) relative to the healthy controls. Similarly, the CC genotype, C allele, and recessive model were observed to be related to the increased incidence of BC with respect to FA (*p* < 0.001 for all). A significant upregulation of HOTTIP and a marked decrease of miR-615-3p were verified in patients with BC compared to each of the healthy individuals, patients with FA, and the non-BC group (healthy subjects + FA) (*p* < 0.001 for all). A significant negative correlation was demonstrated between the expression of HOTTIP and miR-615-3p in the serum of patients with BC. The HOTTIP expression was upregulated, while that of miR-615-3p was downregulated in patients with BC who carried the CC genotype with respect to those who carried the AA or AC genotypes (*p* < 0.05 for all). Conclusions: The genetic variants of rs1859168 are linked to an increased susceptibility to BC. Moreover, HOTTIP and miR-615-3p may be used as novel indicators and targets for the treatment of patients with BC.

## 1. Introduction

Breast cancer (BC) is considered the most frequent malignancy diagnosed among women [1]. Genetic alterations play an essential role in BC pathogenesis [2]. Most breast cancers are diagnosed at a progressive stage due to difficulties in the early detection of the disease. Therefore, detecting new biomarkers is necessary to diagnose BC and predict its behavior, allowing the development of new therapeutic targets [3].

Long noncoding RNAs (lncRNAs) are recognized as RNA molecules with more than 200 nucleotides in length and without a protein-coding capability [4]. Emerging evidence has recently shown that lncRNAs play an essential role in the regulation of some genes concerning the suppression of tumors or oncogenes affecting tumor initiation and progression [5].

The HOXA transcript at the distal tip (HOTTIP) is a lncRNA found at the 5′-end of the HOXA cluster [6]. Numerous works have revealed that HOTTIP regulates HOXA gene expression so that it may play a role in cancer pathogenesis. The upregulation of HOTTIP expression has been described in a variety of malignancies [7]. Interestingly, Yang and his colleagues also reported a positive correlation in BC between HOTTIP expression and lymph node metastasis, tumor size, and TNM staging [8].

However, the prevalence and functional significance of HOTTIP in breast cancer still requires more investigation.

Many recent researchers have revealed that single-nucleotide polymorphisms (SNPs) present in lncRNA genes affect the function and expression of those lncRNAs, representing an important risk factor of disease susceptibility [9]. Furthermore, numerous studies have explained the associations between lncRNA polymorphisms and the susceptibility to different cancers [10,11]. Recently, Hu et al. identified that the functional variant rs1859168 (A/C) in the HOTTIP regulation region contributed to the risk of pancreatic cancer [12], considering A as the wild allele and C as the mutant one. However, there were no studies that reported the associations between this SNP and BC risk.

MicroRNAs (miRNAs) are small noncoding RNAs (19–25 nucleotides in length). Accumulating evidence has linked miR-615-3p to many cancers, it was revealed that miR-615-3p was downregulated in hepatoma cells and breast cancer cells, as well as pancreatic ductal adenocarcinoma [13,14,15]. Additionally, Bai et al. verified that miR-615 was significantly repressed in BC tissues and inhibited the expression of AKT2 [14]. However, little is known about its role in BC. Moreover, it was proven that HOTTIP directly targets miR-615 (as it acts as a competing endogenous RNA (ceRNA) for miR-615-3p, resulting in the activation of its endogenous targets), affecting the growth of renal cell carcinoma [16]. 

In the current study, we aimed to evaluate whether the rs1859168 SNP is associated with breast cancer or the development of fibro adenoma (FA). Additionally, we performed this work to find out whether this polymorphism could affect the serum expression level of HOTTIP and its target microRNA (miR-615-3p) and explore any correlations with the clinicopathological features. Furthermore, we investigated the possibility of using HOTTIP and miR-615-3p as noninvasive biomarkers of BC, analyzing the correlations between their serum expression levels and pathology, as well as the clinical data in BC. 

## 2. Materials and Methods

### 2.1. Subjects

The current study included 151 BC patients who were histologically or cytologically confirmed to have BC. Additionally, 139 female patients with FA were incorporated into this study. Patients were recruited from the outpatients and inpatients of the Surgery Department, Faculty of Medicine, Fayoum University and from The National Cancer Institute, Cairo University during the period from December 2018 to May 2020.

Patients with a history of any other malignancies, current infectious or inflammatory conditions, or those who received any direct or specific treatments for cancer were excluded from the study.

A total of 143 healthy age-matched women who underwent a medical examination in the outpatient clinic of the Surgery Department, Faculty of Medicine, Fayoum University volunteered as the control subjects. Neither they nor their families had a history of cancer.

All the enrolled participants gave signed informed consent. Ethical approval was obtained from the Faculty of Medicine, Fayoum University Local Ethics Committee, which was in-line with the ethical guidelines of the Declaration of Helsinki. 

### 2.2. Blood Sample Processing

Venous blood samples were taken from each participant. Samples were delivered into a plain vacutainer with a gel separator. The blood was allowed to clot for 15 min, and the serum was separated by centrifugation at 4000× *g* for 10 min. Serum samples were immediately stored at −80 °C until use. Another group of whole-blood samples was drawn in EDTA-containing tubes for the DNA extraction and genotyping of rs1859168.

### 2.3. Total RNA Extraction and Reverse Transcription

MiRNeasy extraction kit (Qiagen, Hilden, Germany) was used to get the total RNA (including microRNAs and lncRNAs) extracted from the serum samples after adding the QIAzollysis reagent following the manufacturer’s protocol. The extracted RNA was determined using the NanoDrop^®^ (ND)-1000 spectrophotometer (NanoDrop Technologies Inc., Wilmington, DE, USA).

Total RNA was reverse-transcribed using the RT2 first strand Kit (Qiagen, Maryland, MY, USA) in a whole volume of 20 μL/reaction for the long noncoding RNA analysis, while the miScript II RT kit (Qiagen) was used for the miRNA analysis in a 20-μL RT reaction according to the manufacturer’s instructions.

### 2.4. HOTTIP and miR-615-3p Detection by RT-qPCR

RT-qPCR of the HOTTIP expression was performed using the RT2 SYBR Green PCR kit (Qiagen, Maryland, MY, USA). The expression of miR-615-3p was quantified using the miScript SYBR Green PCR kit (Qiagen, Valenica, CA, USA) following the instructions of the manufacturer. The RefSeq accession no. of HOTTIP was NR_037843.3, and the catalog number of miR-615-3p was MS00029225. All the reactions were done in the Rotor gene Q System (Qiagen) on a 20-μL reaction mixture with the following settings for the HOTTIP assessment: 95 °C for 10 min; subsequently, 40 cycles at 95 °C for 15 s and 60 °C for 60 s. However, for the assessment of miR-615-3p, the cycling conditions were as follows: 95 °C for 30 min, followed by 40 cycles at 94 °C for 15 s, 55 °C for 30 s, and 70 °C for 30 s.

The expression values of HOTTIP were normalized using GAPDH as an endogenous reference gene [17,18]. However, for the calculation of the miR-615-3p expression level, SNORD 68 was used as an internal control. The GAPDH primer sequences were forward 5′-CCCTTCATTGACCTCAACTA-3′ and reverse 5′-TGGAAGATGGTGATGGGATT-3′. The catalog number of SNORD 68 was MS00033712. The equation 2^−ΔΔCt^ was used to calculate the fold changes (FC) of HOTTIP and miR-615-3p [19]. The FC of the healthy group was assumed as 1.

### 2.5. DNA Extraction and Genotyping

Genomic DNA was extracted from whole blood using a Qia-amplification DNA extraction kit (Qiagen, Valenica, CA, USA) following the manufacturer’s instructions. A NanoDrop^®^ (ND)-1000 spectrophotometer (NanoDrop Technologies Inc., Wilmington, DE, USA) was used to assess the quantitation and purity of the DNA samples. The genotyping of rs1859168 (A/C) of HOTTIP (PN4351379) was performed using a predesigned TaqMan SNP genotyping assay (Applied Biosystems, Thermo Fisher Scientific, Foster City, CA, USA), in agreement with the instructions of the manufacturer. RT-PCR was done on a Rotor gene Q System (Qiagen, Valenica, CA, USA). The cycling conditions included denaturation for 10 min at 95 °C, 45 cycles at 92 °C for 15 s, and 60 °C for 90 s for annealing and extension.

### 2.6. Statistical Analysis 

The Statistical Package for Social Sciences (SPSS) version 24 (USA) was used in performing the statistical analysis. The representation of the quantitative data was done by using the mean, standard deviation (SD), standard error of mean (SEM), median, and interquartile range (IQR). For the categorical data, a chi-square test was done. Meanwhile, the continuous variables were analyzed via the Mann–Whitney *U* test, which were presented as medians (interquartile range). Odds ratios (ORs) with 95% confidence intervals (CI) (which were age-adjusted) of alleles and genotypes frequency of rs1859168 in the studied groups were assessed using the multivariate logistic regression analysis. Nonparametric data and categorical data were compared using the chi-square test. Spearman’s correlation was run to determine the correlation between HOTTIP and miR-615-3p.

Receiver operating characteristic (ROC) curve analyses were performed to conclude the sensitivity and specificity of HOTTIP and miR-615-3p as predictors in discriminating BC from other groups. *p*-values less than 0.05 were considered statistically significant.

## 3. Results

### 3.1. Demographic, Clinical, and Pathological Features of the Participants

The detailed findings of the general characteristics of the study participants in the current work are clarified in Table 1.

No marked differences were noted concerning the mean age among the patients with BC or FA when compared to the healthy group (*p* > 0.05). Similarly, no marked differences were noted regarding the ages between the BC and FA groups (*p* > 0.05).

Significant statistical differences were observed among the BC patients and those with FA and between the BC patients compared with the controls regarding the percentage of positive family history of BC (elevated in the BC group, *p* < 0.001 for both).

Considering the clinicopathological data, 117 (77.48%) of all patients presented with invasive duct carcinoma (Grade 2) and 28 (18.55%) with invasive duct carcinoma (grade 3), while only six patients (3.97%) were diagnosed with invasive lobular carcinoma. For the TNM staging, the percentage of patients with T 2 and 3 were 58.94% and 41.06%, respectively, while the percentage of patients with N1, 2, and 3 were 14.57%, 58.28%, and 27.15%, respectively. Concerning the TNM staging, most patients (86.09%) were grade three, whereas 13.91% were grade two. Regarding the estrogen and progesterone receptors (ER/PR), 35 (23.18%) patients were positive for ER/PR, and 116 (67.82%) were negative for ER/PR. Finally, 52.31% of the tumors were smaller than 5cm, while 47.69% were larger than 5 cm.

### 3.2. Frequency Distribution of the Alleles and Genotypes of rs1859168 in Patients with BC, FA, and Healthy Subjects

The distribution of the genotypes of rs1859168 in the control individuals obeyed the Hardy-Weinberg equilibrium (*p* = 0.570).

The distribution of the different genotypes and alleles of rs1859168 are shown in Table 2. Comparing BC patients with the healthy group, we revealed that the CC genotype (considering the AA genotype as a reference) was linked strongly to a high incidence of breast cancer (adjusted OR = 1.983, 95% CI: 0.987–4.572, *p* = 0.013). However, the AC genotype was significantly associated with a low incidence of BC (adjusted OR = 8.534, 95% CI: 2.478–17.392, *p* < 0.001).

Considering the dominant model, when the AA genotype was taken as a reference, the AC + CC genotypes were considerably associated with an increased BC susceptibility (adjusted OR = 5.271, 95% CI: 1.223–13.130, *p* < 0.001). Additionally, in the recessive model, the CC genotype was associated with an increased risk of BC (adjusted OR = 7.588, 95% CI: 5.33–14.478, *p* < 0.001). In addition, the C allele was associated with a high risk of BC (adjusted OR = 9.52, 95% CI: 4.823–19.408, *p* < 0.001).

More importantly, comparing patients with BC against those with FA, the CC genotype proved to be strongly associated with an increased incidence of BC (adjusted OR = 6.015, 95% CI: 2.557–7.916, *p* < 0.001), while the AC genotype was revealed to be significantly low in patients with BC (adjusted OR = 4.25, 95% CI: 1.56–10.096, *p* < 0.001). Besides, when considering the recessive model, rs1859168 polymorphism was associated with a high risk of BC (adjusted OR = 3.251, 95% CI: 1.072–8.364, *p* < 0.001). In addition, the frequency of the C allele was notably elevated in BC with respect to FA patients (adjusted OR = 5.83, 95% CI: 3.16–9.912, *p* < 0.001).

However, no statistical differences were present between the patients with FA and the controls regarding the genotypes and allelic distribution of rs1859168, except that the CC genotype was associated with a low risk of FA (adjusted OR = 2.851, 95% CI: 0.954–5.02, *p* = 0.023).

### 3.3. HOTTIP and miR-615-3p Expression Levels in the Serum of Patients with BC, FA, and Healthy Controls

**Figure 1:**
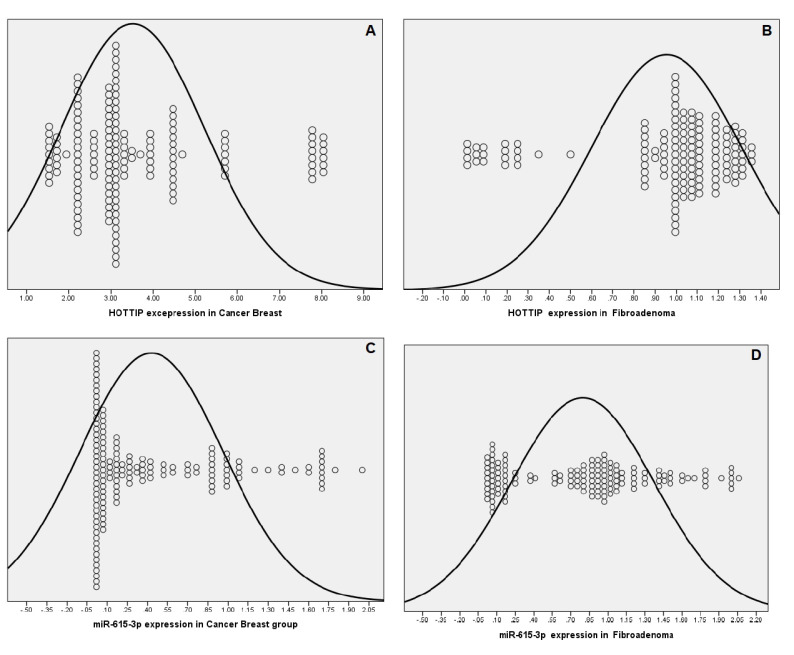
Dot plot representation of the relative expression serum levels of HOTTIP and miR-615-3p in all the study subjects: (**A**) Relative expression of the fold change of the serum HOTTIP in breast cancer patients. (**B**) Relative expression of the fold change of the serum HOTTIP in fibroadenoma patients. (**C**) Relative expression of the fold change of the serum miR-615-3p in breast cancer patients. (**D**) Relative expression of the fold change of the serum miR-615-3p in fibroadenoma patients.

The expression levels of HOTTIP and miR-615-3p in the different studied groups are shown in Figure 1.

HOTTIP had a higher expression (median FC (IQR) was 3.05 (0.09–10.20)), while miR-615-3p had a lower expression (median FC (IQR) was 0.19 (0.004–2.00)) in the serum of BC patients compared to the healthy ones (*p* < 0.001 for HOTTIP and miR-615-3p). In the same way, patients with BC had a significantly increased level of HOTTIP and a significantly decreased level of miR-615-3p relative to patients with FA and, also, relative to the non-BC group (healthy subjects + FA) (*p* < 0.001 for all). Through the comparison of the FA cases and control subjects, no marked differences were documented regarding the serum levels of HOTTIP and miR-615-3p (median FC (IQR) of HOTTIP was 1.01 (0.85–1.36), while that for miR-615-3p was 0.89 (0.02–2.06)).

### 3.4. The Influence of rs1859168 on the Serum Levels of HOTTIP and miR-615-3p in Patients with BC and FA

We investigated whether rs1859168 was associated with the expression of HOTTIP and miR-615-3p in patients with BC and FA.

Of note, patients with BC who carried the rs1859168 CC genotype expressed a significantly higher level of HOTTIP (median FC was 4.04, *p* < 0.001) and a significantly lower level of miR-615-3p (median FC was 0.11, *p* < 0.01) compared with those who carried AA (median FC was 2.04 for the HOTTIP expression, and the median FC was 0.56 for miR-615-3p). Moreover, HOTTIP was markedly upregulated (median FC was 4.04, *p* < 0.01), while miR-615-3p was markedly downregulated (median FC was 0.11, *p* < 0.01) in BC patients carrying the CC genotype with respect to those with the AC genotype (median FC was 3.1 for the HOTTIP expression, and the median FC was 0.35 for miR-615-3p). In addition, the HOTTIP was drastically elevated in patients with BC who carried the AC genotype compared to those carrying the AA genotype (*p* = 0.05). The C allele was associated with a higher expression level of HOTTIP and a lower expression of miR-615-3p relative to the A allele in patients with BC (median FC was 3.5, *p* < 0.001 for HOTTIP, and the median FC was 0.19, *p* = 0.004 for miR-615-3p).

**Figure 2:**
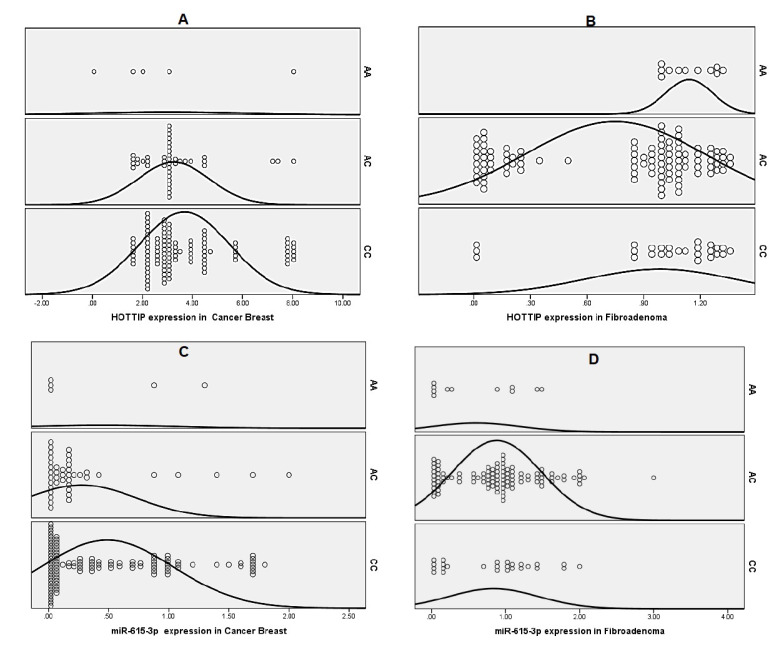
Dot plot representation of the effect of HOTTIP (rs1859168) polymorphism on the expression of serum HOTTIP and miR-615-3p in patients with BC and FA. (**A**) Relative expression of the fold change of the serum HOTTIP in different genotypes of rs1859168 in patients with breast cancer. (**B**) Relative expression of the fold change of the serum HOTTIP in different genotypes of rs1859168 in patients with fibroadenoma. (**C**) Relative expression of the fold change of the serum miR-615-3p in different genotypes of rs1859168 in patients with breast cancer. (**D**) Relative expression of the fold change of the serum miR-615-3p in different genotypes of rs1859168 in patients with fibroadenoma.

Conversely, no significant differences were detected regarding the expression of HOTTIP and miR-615-3p in different genotypes of rs1859168 in patients with FA (Figure 2).

### 3.5. Associations between the rs1859168 Genotypes, Alleles, Laboratory Data, and Clinical and Pathological Features of Patients with BC

Next, we assessed the relations between the different genotypes and alleles of rs1859168 and the clinicopathological data among the BC cases (Table 3).

The incidence of the CC genotype and C allele was increased in individuals who had a positive family history of BC compared with the AC + AA genotype or A allele, respectively (adjusted OR = 3.307, 95% CI: 1.982–6.148, *p* = 0.02 and adjusted OR = 2.359, 95% CI: 1.152–3.558, *p* = 0.04, respectively).

Regarding the T classification of TNM staging, BC patients with the CC genotype and/or the C allele were more at risk of developing T3 than those with the AC + AA genotype or A allele, respectively (adjusted OR = 1.334, 95% CI: 0.886–3.442, *p* = 0.05 and adjusted OR = 2.462, 95% CI: 1.95–6.087, *p* = 0.007, respectively). 

Compared with the A allele, the frequency of the C allele was markedly related to developing N2 and N3 of TNM staging with respect to N1 (adjusted OR = 2.335, 95% CI: 1.85–5.553, *p* = 0.008 and adjusted OR = 1.86, 95% CI: 0.943–3.421, *p* = 0.05, respectively). 

Interestingly, BC patients carrying the C allele were at more risk of developing stage III than stage II, according to the TNM staging (adjusted OR = 2.087, 95% CI: 1.246–6.02, *p* = 0.01). In addition, compared with the A allele, the C allele was significantly associated with ER/PR positivity (adjusted OR = 1.258, 95% CI: 0.689–3.08, *p* = 0.03).

### 3.6. HOTTIP and miR-615-3p Expression Levels in Relation to the Pathological Features and Clinical Parameters in Patients with BC

Table 4 shows the median expression of HOTTIP and miR-615-3p in BC patients classified by different clinicopathological features.

There was a significant overexpression of HOTTIP and downregulation of miR-615-3p in patients with BC whose ages ≥35 (*p* = 0.04 and *p* = 0.003, respectively). Moreover, there was a considerable elevation of the HOTTIP level and a significant decrease in miR-615-3p in patients with BC with a positive family history, as well as in patients who had positive ER/PR (*p* < 0.05). Besides, a marked decrease in the miR-615-3p expression was noted in the present research in patients who developed grade III diseases according to the TNM staging than those with grade II (*p* = 0.05).

In contrast, no marked association was detected between the HOTTIP or miR-615-3p expression levels and other clinicopathological features.

### 3.7. Correlation between the Serum HOTTIP and miR-615-3p Levels in Patients with BC

**Figure 3:**
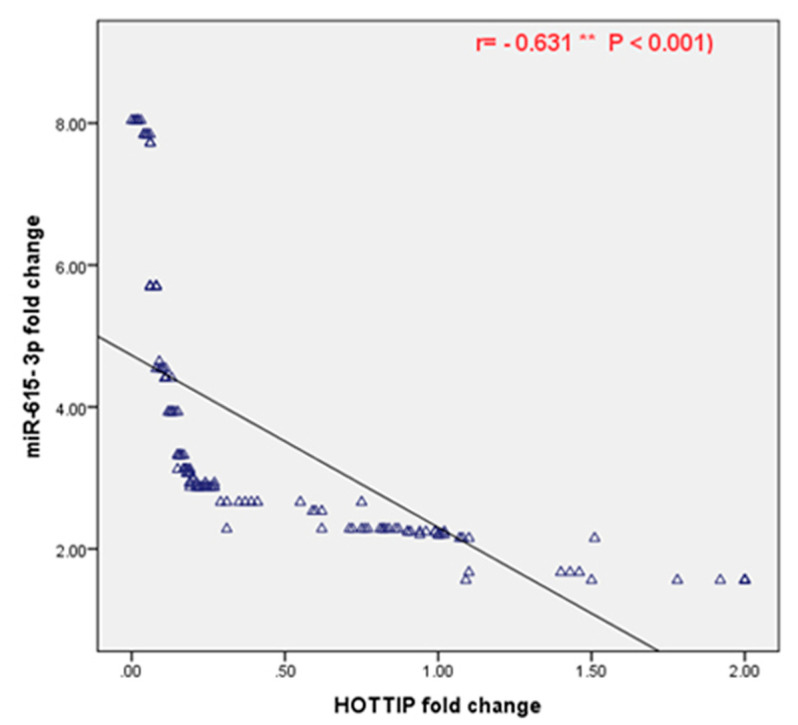
Spearman correlation between the HOTTIP and miR-615-3p serum expression levels in patients with BC. ** Significant at *p* < 0.001.

The Spearman analysis revealed a negative correlation between the expression level of HOTTIP and miR-615-3p in patients with BC (r = −0.631 and *p* < 0.001) (Figure 3).

### 3.8. Evaluation of Serum HOTTIP and miR-615-3p as Diagnostic Biomarkers of BC

**Figure 4:**
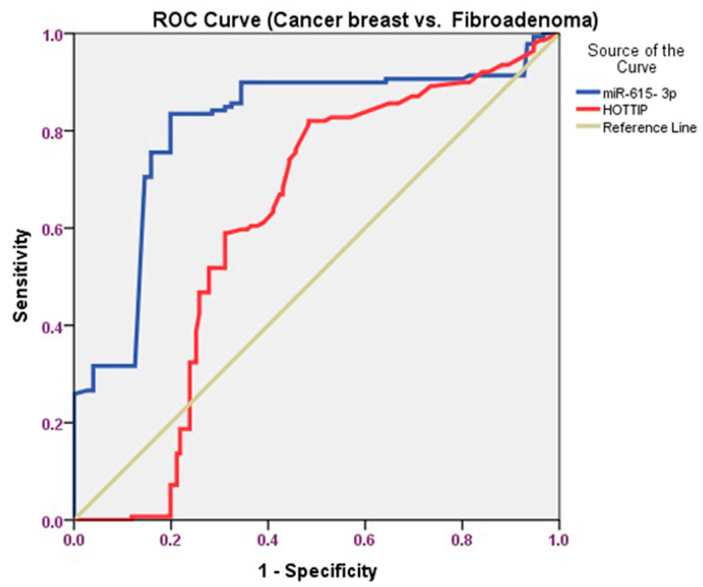
ROC curve analysis of the serum HOTTIP and miR-615-3p for differentiating BC patients from patients with FA.

To assess the accuracy of the serum HOTTIP and miR-615-3p for the early detection of BC, we conducted receiver operating characteristic (ROC) curves to distinguish BC patients from those with FA. For the serum HOTTIP, the AUC was 0.613 (95% confidence interval: 0.545–0.680), and the sensitivity and specificity were 91.2% and 87.95%, respectively. Likewise, the AUC concerning the serum miR- 615-3p was 0.816 (95% confidence interval: 0.763–0.869), with a sensitivity and specificity of 89.8% and 98.5%, respectively (Table 5 and Figure 4).

**Figure 5:**
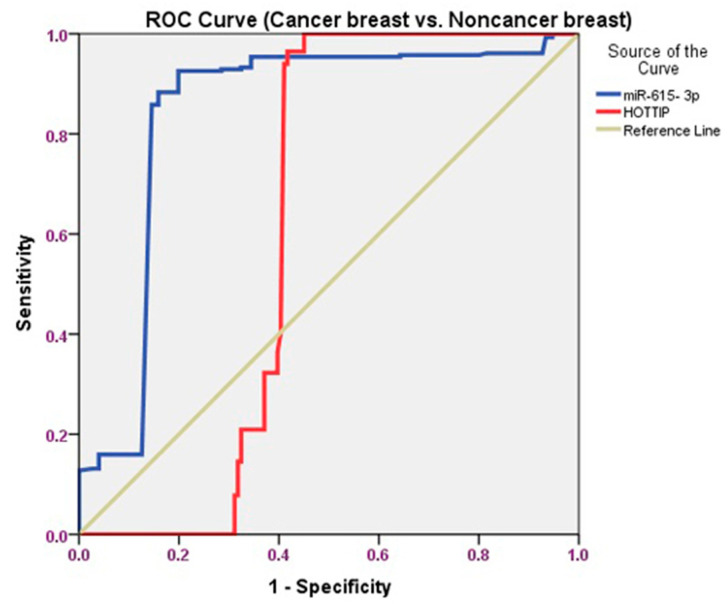
ROC curve analysis of the serum HOTTIP and miR-615-3p for distinguishing patients with BC against those without BC (FA+ healthy persons).

A ROC curve was performed to find out whether the serum HOTTIP and miR-615-3p could differentiate BC from those without BC (FA+ healthy persons). Concerning HOTTIP, the AUC was 0.615 (95% confidence interval: 0.540–0.689), and the sensitivity and specificity were 94.5% and 89.5%, respectively. Furthermore, miR-615-3p showed an AUC of 0.841 (95% confidence interval: 0.793–0.888), and the sensitivity and specificity were 90.4% and 95.6%, respectively (Table 5 and Figure 5).

## 4. Discussion

Breast cancer is one of diseases that occurs frequently in Egypt [20]. The early diagnosis of BC is associated with a low mortality rate [21]. Surgery is valuable in treating early BC, though recurrence might occur in several patients [22]. Therefore, discovering novel noninvasive biomarkers has become an urgent necessity to help with an early diagnosis and to develop novel therapeutic targets.

MiRNAs and lncRNAs are two classes of noncoding RNAs verified to play a necessary role in the pathogenesis of numerous cancers, including BC [9,13]. Furthermore, the evaluation of noncoding RNAs in the serum has emerged as a novel noninvasive biomarker of BC [23,24]. To date, many studies have suggested that lncRNA polymorphisms could alter the function and expression of those lncRNAs influencing the susceptibility to cancers [10,11]. However, the association of rs1859168 in HOTTIP with BC susceptibility has not been addressed yet.

In this study, we revealed that the CC genotype and C allele were strongly linked to an increased risk of BC compared with the control or FA groups, while the AC genotype was associated with a decreased incidence of BC relative to healthy individuals or patients with FA.

In recent years, few researchers have reported an association between rs1859168 in HOTTIP and the risk of cancers. Rs1859168 was hypothesized to modulate the expression of HOTTIP through influencing the transcription factor-binding sites. In addition, rs1859168 was reported to affect the folding and function of HOTTIP [25].

Gong et al. revealed that individuals who carry the AA genotype might be at a decreased risk for lung cancer [25]. Additionally, Ali et al. demonstrated a significant association between rs1859168 polymorphism (CC genotype and C allele) and susceptibility to colorectal cancer in the Egyptian population, which is similar to our results [26].

In contrast, it was observed that rs1859168 polymorphism in HOTTIP was associated with a decreased risk of pancreatic cancer; the authors found that the C allele of rs1859168 was markedly related to a reduced susceptibility to PC [12]. Furthermore, Duan and his colleagues suggested that the HOTTIP SNP rs1859168 was notably associated with a reduced gastric cancer susceptibility in the Chinese population [9]. However, the rs1859168 polymorphism was not related to the incidence of ovarian cancer in the research that was performed by Richards et al. [27].

In our study, we showed that the HOTTIP level is markedly upregulated, while miR-615-3p is markedly reduced in the serum of patients with BC compared to the control individuals or patients with FA.

Our findings were in-line with the results of Sun et al., who determined that HOTTIP is significantly upregulated in the breast cancer cell line and related to BC proliferation, migration, and apoptosis via the modulation of the expression of HOXA11 [28]. Similarly, it was documented that the BC tissue samples had elevated expression levels of HOTTIP, and they were associated with a grim prognosis [8].

In previous studies, it has been verified that the knockdown of HOTTIP resulted in reduced cell proliferation and attenuated metastasis in hepatocellular carcinoma and in non-small cell lung cancer [29,30]. Additionally, two other studies showed the association between the elevated expression level of HOTTIP and each of dismal prognosis and metastasis in PC and hepatocellular carcinoma [31,32].

Accumulating evidence has explained that lncRNAs have a major role in tumor progression via targeting miRNAs [33]. It was shown that HOTTIP binds to miR-615, resulting in its downregulation [16,34]. Increasing studies have indicated the implication of miR-615 in numerous cancers, as it was verified to be a tumor suppressor [13,14,15]. However, to the best of our knowledge, our study is the first to assess the miR-615-3p expression in the serum of patients with BC.

The results from our series are in agreement with Bai et al., who revealed that miR-615 was significantly repressed in BC tissues and inhibited the expression of AKT2 [14]. However, the upregulation of miR-615-3p in BC cells was shown. It was elucidated that miR-615-3p promotes the metastasis and epithelial-mesenchymal transition through targeting Protein Interacting with C Kinase -1 (PICK1) [35]. Interestingly, the miR-615-3p expression level proved to be increased in the luminal B HER 2-positive cases, while decreased in HER2-enriched BC [36].

More importantly, our results indicated that there is a negative correlation between the serum expression level of HOTTIP and that of miR-615-3p. Consistent with our hypothesis, Wang et al. reported a negative correlation between the aforementioned noncoding RNAs in renal carcinoma [16]. Additionally, Shi and his colleagues confirmed that HOTTIP is negatively correlated to miR-615-3p in non-small cell lung cancer cells [34].

The polymorphisms in lncRNAs not only influence the levels of mature lncRNAs but also affect their target gene expression. Therefore, we analyzed, for the first time, whether HOTTIP and miR-615-3p were changed significantly among the different genotypes of rs1859168 in patients with BC.

We determined that the CC genotype and C allele were related to an elevated expression of HOTTIP and a decreased expression of miR-615-3p in BC.

In contrast, the levels of HOTTIP and miR-615-3p were not significantly changed among the different genotypes and allele distribution in FA. This is in contract with the study performed by Hu et al., in which the C allele of rs1859168 was determined to be related to the decreased levels of HOTTIP in PC [12].

Our current study reported that BC patients with the CC genotype and or C allele (which were related to an elevated expression of HOTTIP) were more at risk of developing T3 than those with the AC + AA genotype or A allele, respectively. Furthermore, the frequency of the C allele was markedly related to developing N2 and N3 of TNM staging with respect to N1. In addition, BC patients carrying the C allele were more at risk of developing stage III than stage II, according to the TNM staging.

Our results are in agreement with previous research that demonstrated the association between high levels of HOTTIP with a bad prognosis and advanced TNM staging in BC [8]. Similarly, Wang et al. verified that upregulated HOTTIP is related to a higher clinical stage, tumor size, metastasis lymph node, and worse clinical stage in renal cell carcinoma [16].

The ROC curve was carried out and proved that serum HOTTIP could be used to discriminate patients with BC from the FA group and those without BC (FA+ healthy persons).

Future studies are needed to clarify the mechanism of how rs1859168 influences BC pathogenesis, as well as to explain its links with HOTTIP and miR-615-3p expression in other ethnic groups. In addition, the exact role of the aforementioned noncoding RNAs in BC should be investigated and clarified. The present results should be confirmed in further, larger research.

To sum up, our current findings indicated that the rs1859168 genetic variants were related to the increased incidence of BC. Additionally, our results suggested that serum HOTTIP and miR-615-3p may have indicative potential to predict BC and may be used as targets for BC treatment. Furthermore, we recognized a negative correlation between HOTTIP and miR-615-3p in the serum of patients with BC. Finally, we verified that the CC genotype and C allele were associated with high levels of HOTTIP and low levels of miR-615-3p in patients with BC.

## Figures and Tables

**Table 1:** Characteristics of the participants.

Variables		BC (*n* = 151)	FA (*n* = 139)	Control (*n* = 143)	*p*-Value (BC vs. control)	*p*-Value (BC vs. FA)	*p*-Value (FA vs. control)
Age (years)	Mean ± SD	54.91 ± 6.43	51.08 ± 9.73	49.89 ± 10.37	0.09	0.341	0.178
Family history	Yes (BC) *n* (%)	94 (62.25)	2 (1.44)	1 (0.70)	<0.001 *	<0.001 *	0.19
	Yes (FA) *n* (%)	15 (9.93)	11 (7.91)	2 (1.40)	0.06	0.09	0.15
	No *n* (%)	42 (27.81)	126 (90.65)	140 (97.90)	<0.001 *	0.01 *	0.182
History of Hypertension	Yes *n* (%)	25 (16.56)	10 (7.19)	8 (5.59)	0.54	0.243	0.821
	No *n* (%)	126 (83.44)	129 (92.81)	135 (94.41)	0.214	0.343	0.88
History of DM	Yes *n* (%)	29 (19.21)	22 (15.83)	15 (10.49)	0.17	0.69	0.54
	No *n* (%)	122 (80.79)	117 (84.17)	128 (89.51)	0.39	0.67	0.24
Menstrual history	Premenopausal (regular)	24 (15.89)	51 (36.69)	55 (38.46)	0.09	0.08	0.401
	Premenopausal (irregular)	26 (17.22)	41 (29.50)	53 (37.06)	0.19	0.06	0.108
	Postmenopausal	101 (66.89)	47 (33.81)	35 (24.48)	0.06	0.09	0.28
Hormonal Contraceptive	Yes *n* (%)	103 (68.21)	89 (64.03)	67 (46.85)	0.13	0.53	0.18
	No *n* (%)	48 (31.79)	50 (35.97)	76 (53.15)	0.49	0.56	0.27
Type of tumor *n* (%)	Invasive Duct ii	117 (77.48)					
	Invasive Duct iii	28 (18.55)					
	Invasive Lobular Carcinoma	6 (3.97)					
T Classification *n* (%)	T2	89 (58.94)					
	T3	62 (41.06)					
N Classification *n* (%)	N1	22 (14.57)					
	N2	88 (58.28)					
	N3	41 (27.15)					
M Classification *n* (%)	Zero	151 (100)					
TNM staging *n* (%)	II	21 (13.91)					
	III	130 (86.09)					
ER/PR *n* (%)	Positive	35 (23.18)					
	Negative	116 (67.82)					
Tumor Size *n* (%)	<5 cm	79 (52.31)					
	>5 cm	72 (47.69)					

BC: Breast Cancer, FA: Fibroadenoma, DM: Diabetes mellitus, and ER/PR: Estrogen Receptor/Progesterone Receptor. Data are expressed as the mean ± (SD) or *n* (%). *p*-values were determined using an independent *t*-test or chi-square test as an appropriate comparison. * Significant at *p* < 0.05.

**Table 2:** Distribution frequency of the rs1859168 genotypes and alleles in patients with breast cancer, fibroadenoma, and healthy subjects.

rs1859168 (A/C)		Control (143)	FA (139)		BC (151)		
		*n* (%)	*n* (%)	OR (95% CI)/*p*-Value (FA vs. Control)	*n* (%)	OR (95% CI)/*p*-Value (BC vs. Control)	OR (95% CI)/*p*-Value (BC vs. FA)
Genotype	AA	49 (34.26)	11 (7.91)	1	5 (3.31)	1	1
	AC	63 (44.06)	113 (81.30)	1.552 (0.129–2.17)/0.08	42 (27.82)	8.534 (2.478–17.392)/<0.001 *	4.25 (1.56–10.096)/<0.001 *
	CC	31 (21.68)	15 (10.79)	2.851 (0.954–5.02)/0.023 *	104 (68.87)	1.983 (0.987–4.572)/0.013 *	6.015 (2.557–7.916)/<0.001 *
Dominant Model	AA	49 (34.26)	11 (7.91)	1	5 (3.31)	1	1
	AC + CC	94 (65.74)	128 (92.09)	1.223 (0.889–2.08)/0.09	146 (96.69)	5.271 (1.223–13.130)/<0.001 *	1.033 (0.795–1.883)/0.208
Recessive Model	CC	31 (21.68)	15 (10.79)	0.98 (0.241–2.422)/0.791	104 (68.87)	7.588 (5.33–14.478)/<0.001 *	3.251 (1.072–8.364)/<0.001 *
	AC + AA	112 (78.32)	124 (89.21)	1	47 (31.13)	1	1
Allele	A	161 (56.29)	135 (48.56)	1	47 (15.65)	1	1
	C	125 (43.71)	143 (51.44)	1.097 (0.015–1.731)/0.196	255 (84.35)	9.52 (4.823–19.408)/<0.001 *	5.83 (3.16–9.912)/<0.001 *

OR: odds ratio and CI: confidence interval. *p*-value is adjusted for age. * Significant at *p* < 0.05.

**Table 3:** Genotype and allele frequencies of rs1859168 according to the clinical characteristics of the patients with BC.

Parameters	CC	AC + AA	OR (95% CI), *p*-Value	C	A	OR (95% CI), *p*-Value
	*n* (%)	*n* (%)		*n* (%)	*n* (%)	
Age						
<35	48 (46.15)	25 (53.19)	1	98 (32.45)	27 (8.94)	1
≥35	56 (53.85)	22 (46.81)	0.522 (0.254–1.954), 0.301	157 (50.99)	20 (6.62)	0.339 (0.187–1.09), 0.661
Family history						
No	29 (27.88)	27 (57.45)	1	188 (62.25)	22 (7.28)	1
Yes	75 (72.12)	20 (42.55)	3.307 (1.982–6.148), 0.02 *	67 (22.20)	25 (8.27)	2.359 (1.152–3.558), 0.04 *
History of HTN						
No	89 (85.85)	37 (78.72)	1	137 (45.36)	19 (6.29)	1
Yes	15 (14.15)	10 (27.28)	0.528 (0.175–0.899) 0.537	118 (39.07)	28 (9.28)	0.562 (0.357–1.076), 0.09
History of DM						
No	87 (83.65)	39 (82.98)	1	146 (48.35)	30 (9.93)	1
Yes	17 (16.35)	8 (17.12)	0.492 (0.119–0.741) 0.357	109 (36.09)	17 (5.63)	0.341 (0.048–0.834) 0.62
Menstrual history						
Pre. (regular)	10 (9.62)	14 (29.79)	1	31 (10.26)	14 (4.63)	1
Pre. (irregular)	15 (14.42)	11 (23.41)	0.293 (0.108–0.458) 0.803	42 (13.91)	8 (2.65)	0.338 (0.147–0.695) 0.233
Postmenopausal	79 (75.96)	22 (46.80)	1.52 (0.684–3.01) 0.05 *	182 (60.27)	25 (8.28)	0.553 (0.277–0.830) 0.426
Contraception						
No	35 (33.65)	13 (27.66)	1	98 (32.45)	15 (4.97)	1
Yes	69 (66.34)	34 (82.34)	0.553 (0.179–0.893) 0.558	157 (51.99)	32 (10.59)	0.835 (0.312–1.078) 0.391
Type Of Tumor						
Invasive Duct II	79 (75.96)	38 (80.85)	1	205 (67.88)	30 (9.94)	1
Invasive Duct III	20 (19.23)	8 (17.02)	0.298 (0.113–0.449) 0.469	43 (14.24)	12 (3.97)	0.229 (0.173–0.501) 0.372
Invasive Lobular II	5 (4.81)	1 (2.13)	0.187 (0.097–0.384) 0.834	7 (2.32)	5 (1.65)	0.308 (0.119–0.486) 0.421
T classification						
T2	59 (56.73)	30 (63.83)	1	118 (39.07)	21 (6.96)	1
T3	45 (43.27)	17 (36.17)	1.334 (0.886–3.442) 0.05 *	137 (45.36)	26 (8.61)	2.462 (1.95–6.087) 0.007 *
N classification						
N1	11 (10.57)	11 (23.40)	1	28 (9.27)	15 (4.97)	1
N2	54 (51.93)	34 (72.35)	0.852 (0.259–1.097) 0.09	123 (40.73)	20 (6.62)	2.335 (1.85–5.553) 0.008 *
N3	39 (37.50)	2 (4.25)	0.519 (0.456–0.82) 0.13	104 (34.44)	12 (3.97)	1.86 (0.943–3.421) 0.05 *
TNM staging						
II	(87.69)	13 (27.65)	1	23 (7.61)	32 (10.60)	1
III	96 (92.31)	34 (72.35)	2.36 (1.941–4.58)	232 (76.82)	15 (4.97)	2.087 (1.246–6.02) 0.01 *
ER/PR						
Positive	25 (24.04)	10 (21.28)	1	77 (25.50)	15 (4.97)	1
Negative	79 (75.96)	37 (78.72)	0.899 (0.379–1.09) 0.175	178 (58.94)	32 (10.59)	1.258 (0.689–3.08) 0.03 *
Tumor Size						
<5 cm	47 (45.19)	32 (68.08)	1	89 (29.47)	19 (6.29)	1
>5 cm	57 (54.81)	15 (31.92)	0.233 (0.183–0.506) 0.277	166 (54.97)	28 (9.27)	0.324 (0.177–0.526) 0.23

BC: Breast Cancer, FA: Fibroadenoma, DM: Diabetes mellitus, ER/PR: Estrogen Receptor/Progesterone Receptor, OR: odds ratio, and CI: confidence interval. *p*-value is adjusted for age. * Significant at *p* < 0.05.

**Table 4:** Differences in the HOTTIP and miR-615-3p serum expression levels in relation to the clinical and pathological features in BC patients.

Parameters		HOTTIP Median (IQR)	miR-615- 3p Median (IQR)	*p*-Value
Age	<35	3.7 (0.81–8.02)	0.35 (0.02–2.00)	0.04 *^,^^a^, 0.003 *^,^^b^
	≥35	4.02 (0.73–10.1)	0.05 (0.004–1.18)	
Family history	Yes	6.08 (1.02–10.2)	0.14 (0.004–1.76)	<0.001 *^,^^a^, 0.02 *^,^^b^
	No	2.01 (0.14–3.57)	0.52 (0.01–2.00)	
History ofHypertension	Yes	3.1 (0.81–10.05)	0.20 (0.004–0.92)	0.194 ^a^, 0.533 ^b^
	No	4.04(0.07–8.05)	0.27 (0.05–2.00)	
History of DM	Yes	3.87 (0.75–8.15)	1.89 (0.01–0.88)	0.567 ^a^, 0.287 ^b^
	No	3.8 (0.16–10.20)	0.25 (0.004–2.00)	
Menstrual history	Premenopausal (regular)	2.9 (0.09–6.04)	0.27 (0.004–1.92)	0.338 ^a^, 0.09 ^b^
	Premenopausal (irregular)	2.5 (0.185–8.45)	0.19 (0.03–2.00)	
	Postmenopausal	3.08 (0.09–10.06)	0.24 (0.07–1.81)	
Hormonal Contraceptive	Yes	3.1 (0.28–6304)	0.21 (0.004–0.179)	0.433 ^a^, 0.294 ^b^
	No	2.9 (0.98–10.2)	0.18 (0.001–2.00)	
type of tumor	invasive duct ii	3.01 (0.65–7.05)	0.18 (0.004–1.89)	0.663 ^a^, 0.138 ^b^
	invasive duct iii	2.09 (0.93–9.05)	0.20 (0.009–1.92)	
	invasive lobular ii	2.87 (0.73–10.02)	0.25 (0.01–2.00)	
T classification	T2	3.5 (0.99–8.55)	0.19 (0.009–1.89)	0.731 ^a^, 0.197 ^b^
	T3	2.9 (0.08–10.01)	0.22 (0.004–2.00)	
N classification	N1	3.09 (0.82–6.72)	0.18 (0.01–1.89)	0.208 ^a^, 0.342 ^b^
	N2	3.01 (0.19–10.05)	0.24 (0.004–1.92)	
	N3	2.89 (0.97–7.85)	0.22 (0.09–2.00)	
TNM staging	II	3.5 (0.07–10.05)	0.25 (0.01–1.76)	0.70 ^a^, 0.05 *^,^^b^
	III	3.05 (0.99–8.55)	0.18 (0.004–2.00)	
ER/PR	Positive	4.04 (0.09–10.2)	0.18 (0.004–1.53)	0.02 *^,^^a^, 0.03 *^,^^b^
	Negative	1.98 (0.93–6.08)	0.29 (0.09–2.00)	
Tumor Size	<5 cm	2.75 (0.9–8.55)	0.19 (0.004–189)	0.297 ^a^, 0.439 ^b^
	>5 cm	3.90 (1.2–10.2)	0.21 (0.009–2.00)	

BC: Breast Cancer, FA: Fibroadenoma, DM: Diabetes mellitus, ER/PR: Estrogen Receptor/Progesterone Receptor, and IQR: Interquartile range. ^a^: *p*-value for HOTTIP and ^b^: *p*-value for miR-615-3p. * Significant at *p* < 0.05.

**Table 5:** ROC curve of the serum HOTTIP and miR-615-3p.

Variable	AUC (95% CI)	*p*-Value	Sensitivity	Specificity	Total Accuracy
HOTTIP (BC vs. FA)	0.613 (0.545–0.680)	0.001 *	91.2%	87.95%	89.57
miR-615-3p (BC vs. FA)	0.816 (0.763–0.869)	<0.001 *	89.8%	98.5%	94.15
HOTTIP (BC vs. control + FA)	0.615 (0.540–0.689)	<0.001 *	94.5%	89.5%	90.00
miR-615-3p (BC vs. control + FA)	0.841 (0.793–0.888)	<0.001 *	90.4%	95.6%	93

BC: Breast Cancer, FA: Fibroadenoma, AUC: area under the curve, and CI: confidence interval. * Significant at *p* < 0.05.

## Data Availability

The data that support the findings of this study are not available due to patients confidentiality.
